# Facile Synthesis of Triangular and Hexagonal Anionic Gold Nanoparticles and Evaluation of Their Cytotoxicity

**DOI:** 10.3390/nano9121774

**Published:** 2019-12-12

**Authors:** R. M. Tripathi, Sun-Young Yoon, Dohee Ahn, Sang J. Chung

**Affiliations:** 1 School of Pharmacy, Sungkyunkwan University, 2066 Seoburo, Jangan-gu, Suwon, Gyeonggido 16419, Korea; rmtripathi@skku.edu (R.M.T.); youcan26@skku.edu (S.-Y.Y.); ehgml94@naver.com (D.A.); 2 Amity Institute of Nanotechnology, Amity University Uttar Pradesh, Sector 125, Noida 201303, India

**Keywords:** green synthesis, *Erigeron annuus* leaf extract, gold nanoparticles, triangular and hexagonal, cytotoxicity, 3T3-L1 preadipocytes

## Abstract

Comprehension of the shape-dependent properties of gold nanoparticles (AuNPs) could benefit the advancements in cellular uptake efficiency. Spherical AuNPs have generally been used for drug delivery, and recent research has indicated that the cellular uptake of triangular AuNPs was higher than that of spherical ones. Previous reports have also revealed that chemically synthesized AuNPs were cytotoxic. Therefore, we have developed a facile, cost-effective, and environmentally friendly method for synthesizing triangular and hexagonal anionic AuNPs. The zeta potential of the synthesized AuNPs was negative, which indicated that their surface could be easily functionalized with positively charged molecules to upload drugs or biomolecules. Transmission electron microscopy (TEM) images illustrated that the largest particle size of the synthesized quasi-hexagonal AuNPs was 61 nm. The TEM images also illustrated that two types of equilateral-triangular AuNPs were synthesized: One featured sharp and the other rounded corners. The sides of the smallest and largest triangular AuNPs were 23 and 178 nm, respectively. Energy-dispersive X-ray spectra of the green-synthesized AuNPs indicated that they consisted entirely of elemental Au. The cytotoxicity of the green-synthesized AuNPs was evaluated using 3T3-L1 adipocytes. Using cell viability data, we determined that the green-synthesized AuNPs did not exhibit any cytotoxic effects on 3T3-L1 adipocytes.

## 1. Introduction

Gold nanoparticles (AuNPs) have been widely used in the optoelectronics field for electronic and magnetic devices; further, these have been employed as catalysts, antibacterials, colorimetric sensors, and also in drug delivery systems [1,2,3,4,5]. Nanoparticles (NPs) are generally synthesized using chemical methods that involve toxic chemicals, expensive techniques, or inefficient consumption of energy and resources [6]. Chemical methods use reducing and capping agents, such as sodium borohydride, sodium citrate, and sodium dodecyl sulfate; these generate significant environmental and human health problems. Hence, researchers have focused on facile, cost-effective, nontoxic, environmentally friendly, green chemistry methods for synthesizing NPs. Nowadays, researchers have been adopting biological routes for the synthesis of nanomaterials using fungal [1,7,8] and bacterial [9] biomasses as well as plant extracts [10,11,12]. Of these biomaterials, plant extracts have gained the attention of researchers over bacterial and fungal biomasses. This is because they facilitate easier, more cost-effective synthesis methods, which are amenable to industrial scale-up and do not require culture maintenance. 

The physical and chemical properties of AuNPs are shape-dependent [13]. Numerous AuNP morphologies have been reported, such as nanospheres [14], nanocubes [15], and nanorods [16]. Elucidating the influence of the shape of NPs on biological systems could provide valuable information for the development of more effective applications for AuNPs. Few papers have been published on the cellular uptake of AuNPs. Particularly, the cellular uptake of rod-shaped AuNPs by HeLa cells was reported to be lower than that of spherical AuNPs [17]. Au nanospheres and nanocages have been studied to analyze the influence of their shape, size, and surface functional group on cellular uptake [18]. A recent study determined that spherical AuNPs exhibited lower cellular uptake than triangular ones of similar surface areas, and this was less equivocal for HeLa cells than for RAW264.7 ones [19]. It was determined that while the chemically synthesized triangular AuNPs exhibited good cellular uptake, the synthesis method presented inherent drawbacks. Hence, environmentally friendly, facile, and cost-effective methods for the synthesis of AuNPs should be developed.

In this study, we have developed a facile, cost-effective, and environmentally friendly method for the synthesis of triangular and hexagonal AuNPs. *Erigeron annuus* (*E. annuus*) leaf extract was used as a reducing and capping agent for the green synthesis of AuNPs. *E. annuus* leaf extract is a good source of γ-pyranone derivatives, flavonoids, and phenolic acids [20], and these biomolecules play significant roles in the green synthesis of NPs [21,22]. *E. annuus* has been used for the treatment of indigestion, hematuria, enteritis, and epidemic hepatitis [23]. The synthesized AuNPs were negatively charged, which allowed us to easily modify their surface with positively charged molecules. It was found that the positively charged nanoparticles produce more cytotoxic properties in A549 cells as compared to negatively charged particles of a similar shape and size [24]. The green-synthesized triangular and hexagonal AuNPs were used to evaluate their cell viability against 3T3-L1 adipocytes.

## 2. Materials and Methods 

### 2.1. Materials

Gold(III) chloride trihydrate (HAuCl_4_·3H_2_O) was purchased from Sigma-Aldrich (St. Louis, MO, USA). The 3T3-L1 preadipocytes were obtained from the American Type Culture Collection (Manassas, VA, USA). Dulbecco’s modified Eagle’s medium (DMEM) was purchased from Welgene (Gyeongsan-si, South Korea). In addition, deionized (DI) water was used for the green synthesis of AuNPs.

### 2.2. E. annuus Leaf Extract Preparation

*E. annuus* leaves were collected from the Suwon campus of Sungkyunkwan University, Gyeonggido, Republic of Korea. The leaves were washed several times with DI water followed by drying at room temperature to remove the adsorbed water from their surface. After mincing them, 8.5 g leaves were added to a 200 mL Erlenmeyer flask that contained 100 mL DI water. The flask was placed on a magnetic stirrer at 500 rpm and the dispersion was boiled. Then, the mixture was allowed to cool to room temperature. The aqueous dispersion was filtrated using Whatman filter paper to obtain the leaf extract that was used for our study. Lastly, the obtained extract was stored at 4 °C for future use for the green synthesis of AuNPs. 

### 2.3. Green Synthesis of AuNPs

HAuCl_4_·3H_2_O (0.002 M) was dissolved in 10 mL DI water, and the flask containing the solution was placed on a magnetic stirrer at 85 °C and 450 rpm. After 15 min, 2 mL *E. annuus* leaf extract was added to the solution dropwise under continuous stirring, and the reaction was run for 30 min. Au^3+^ was reduced to Au^0^ as indicated by the change in color of the reaction mixture from light yellow to dark red. Surface plasmon resonance (SPR) analysis was performed using ultraviolet–visible (UV–Vis) spectroscopy.

### 2.4. Effect of E. annuus Leaf Extract Concentration on Green Synthesis of AuNPs

The *E. annuus* leaf extract was used as a reducing as well as a capping agent. The effect of the leaf extract concentration on the synthesis of AuNPs was evaluated. Different volumes of *E. annuus* leaf extract in the range 0–2 mL were added to 10 mL HAuCl_4_·3H_2_O (0.002 M). Each reaction mixture was added to an Erlenmeyer flask, which was placed on a magnetic stirrer at 450 rpm. The reactions were carried out for 30 min after the addition of the leaf extract.

### 2.5. Effect of Temperature on Green Synthesis of AuNPs

Temperature plays a significant role in the synthesis of NPs. Hence, we have studied the effect of temperature on the synthesis of AuNPs. A series of experiments were conducted at different temperatures (85, 105, 150, and 200 °C), and the results were analyzed. For a typical reaction, 10 mL HAuCl_4_·3H_2_O (0.002 M) solution was added to an Erlenmeyer flask which was heated to the pre-set temperature. Then, 15 min after the target temperature was reached, 2 mL *E. annuus* leaf extract was added dropwise to the flask. 

### 2.6. Characterisation of Nanoparticles

The SPR of the green-synthesized AuNPs was evaluated using an UV–Vis spectroscopy (UH-5300, Hitachi, Japan) device in the scanning range 400–800 nm. The AuNPs were also analyzed using a dynamic light scattering (DLS; Zetasizer Nano S90, Malvern) apparatus to determine their size distribution profile and zeta potential values. The participation of biological molecules to the synthesis of AuNPs was analyzed using a Fourier-transform infrared (FTIR; FTS 7000, Varian, Australia) spectroscopy in the scanning range 500–4000 nm. The size and shape of the green-synthesized AuNPs were analyzed using a transmission electron microscopy (TEM; JEM-3010, JEOL, Japan) instrument. Furthermore, the elemental composition of the AuNPs was examined using energy-dispersive X-ray spectroscopy (EDX). 

### 2.7. Cytotoxicity Assay

#### 2.7.1. Cell Culture 

The methods used for culturing the 3T3-L1 preadipocytes have been previously described by Cho et al. [25]. The 3T3-L1 preadipocytes were cultured in high glucose DMEM that contained 10% bovine calf serum (BCS; Thermo Fisher Scientific, Seoul, Korea) and antibiotic-antimycotic solution (Welgene). 

#### 2.7.2. Cell Differentiation

The methods used for differentiating the 3T3-L1 preadipocytes have been previously described by Cho et al. [25]. When the 3T3-L1 preadipocytes reached 100% confluence, they were cultured in DMEM that contained 10% fetal bovine serum (FBS; Thermo Fisher Scientific Korea Ltd., Seoul, Korea), 0.5 mM isobutylmethylxanthine (Merck KGaA, Darmstadt, Germany) as antibiotic-antimycotic solution, 1 μM dexamethasone (Sigma-Aldrich, St. Louis, MO, USA), and 5 μg/mL insulin (Merck KGaA, Darmstadt, Germany) for 2 days. The cells were then maintained in DMEM supplemented with 10% FBS, antibiotic-antimycotic solution, and 5 μg/mL insulin for two additional days. This was followed by culturing them in DMEM that contained 10% FBS and antibiotic-antimycotic solution for 4 additional days.

#### 2.7.3. Cell Viability Assay

EZ-Cytox assay kits (DOGEN Bio., Seoul, Korea) were used according to the manufacturer’s instructions for measuring cell viability. Differentiated 3T3-L1 adipocytes were incubated with various concentrations of AuNPs (0, 5, 10, 20, 30, and 60 μg/mL) for 48 h and were subsequently assessed at 450 nm using a VictorTM X4 (PerkinElmer, Waltham, MA, USA) microplate reader.

## 3. Results and Discussion

### 3.1. UV–Vis Spectroscopy Analysis

**Figure 1:**
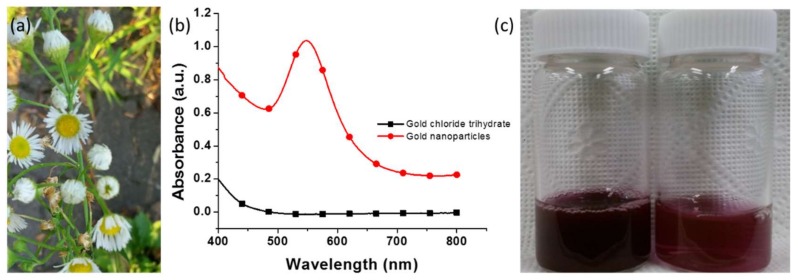
Green synthesis of Au nanoparticles (AuNPs). (**a**) *Erigeron annuus* plants. (**b**) Ultraviolet–visible spectra of green-synthesized AuNPs. (**c**) Dark red-colored dispersion (left vial) of as-synthesized AuNPs and light red-colored dispersion (right vial) of as-synthesized AuNPs diluted with deionized water.

The green-synthesized triangular and hexagonal AuNPs were examined using UV–Vis spectroscopy. The *E. annuus* leaf extract was used as a reducing and capping agent for the synthesis of AuNPs (Figure 1a). After 30 min of incubation, the color of the solution changed from yellow to dark red owing to the SPR effect; this indicated the formation of the AuNPs [3]. Figure 1b illustrates the UV–Vis spectrum of the aqueous HAuCl_4_·3H_2_O solution where no peak was observed at 547 nm. However, an intense peak at 547 nm was observed in the UV–Vis spectrum of the dark red color colloidal dispersion, which corresponded to the SPR of AuNPs [26]. This indicated the capability of the *E. annuus* leaf extract to reduce Au^3+^ to Au^0^. The first vial in Figure 1c (left) contained dark red-colored as-synthesized colloidal AuNP dispersion; the second vial (right) contained red-colored AuNP dispersion after dilution with DI water. The complete reduction of Au^3+^ into Au^0^ occurred within 30 min, whereas previous reports mentioned that 72 h was required to reduce Au^3+^ to Au^0^ using fungal biomass [3]. Therefore, this was a biological method for the rapid synthesis of AuNPs. 

### 3.2. DLS Analysis

**Figure 2:**
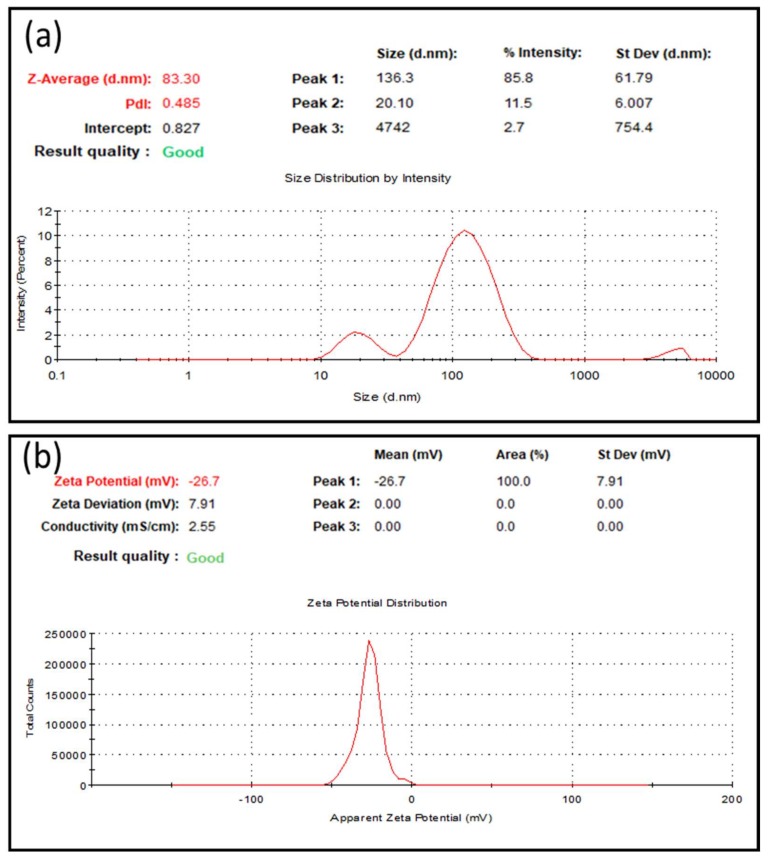
Dynamic light scattering analysis of green-synthesized Au nanoparticles (AuNPs). (**a**) Size distribution profile and (**b**) zeta potential of AuNPs.

The green-synthesized AuNPs were analyzed using DLS to determine their size distribution profile and zeta potential values. Figure 2a depicts the DLS graph of the as-synthesized AuNPs, which illustrates that the Z-average diameter of the AuNPs was 83.3 nm. The polydispersity index is an important parameter for assessing the quality of NPs. The polydispersity indices of good-quality NPs are typically higher than 0.7 [2,27]. The polydispersity index of the AuNPs we synthesized was 0.485 (Figure 2a). The surface charge of the synthesized AuNPs was also determined. The zeta potential of the AuNPs was determined to be −26.7 mV, which indicated that the surface of the AuNPs was negatively charged. This negative potential also indicated the presence of biological moieties on the surface of the synthesized AuNPs, which were responsible for the negative charge.

### 3.3. FTIR Analysis

**Figure 3:**
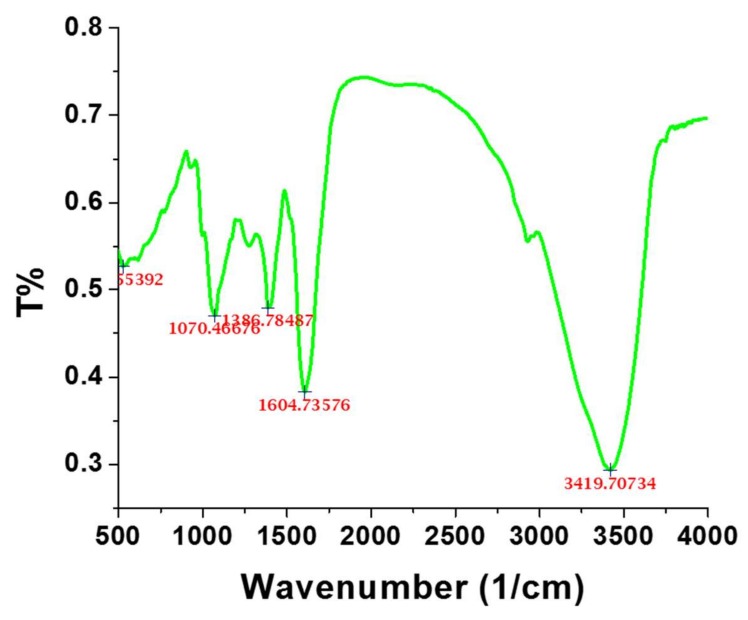
Fourier-transform infrared spectrum of green-synthesized Au nanoparticles.

The FTIR spectrum of the as-synthesized AuNPs was obtained to assess the involvement of the *E. annuus* leaf extract in the synthesis of AuNPs. Figure 3 illustrates the FTIR spectrum of AuNPs in the scanning range of 500–4000 nm. An intense peak was observed at 3419.70 cm^−1^, which corresponded to the –OH stretching vibrations of the OH units and water [11,28]. A second intense peak was observed at 1604.73 cm^−1^, which was ascribed to the local environments of the COO^−^ groups (1610–1550 cm^−1^) in the protein molecule [29]. The peak at 1386.75 cm^−1^ was attributed to the bending of the C–H aldehyde bonds. Lastly, the peak at 1070.46 cm^−1^, corresponded to the flavanones adsorbed on the surface of the AuNPs [30]. The FTIR spectrum indicated that the biomolecules in the *E. annuus* leaf extract were involved in the synthesis of the AuNPs. 

### 3.4. TEM Analysis

**Figure 4:**
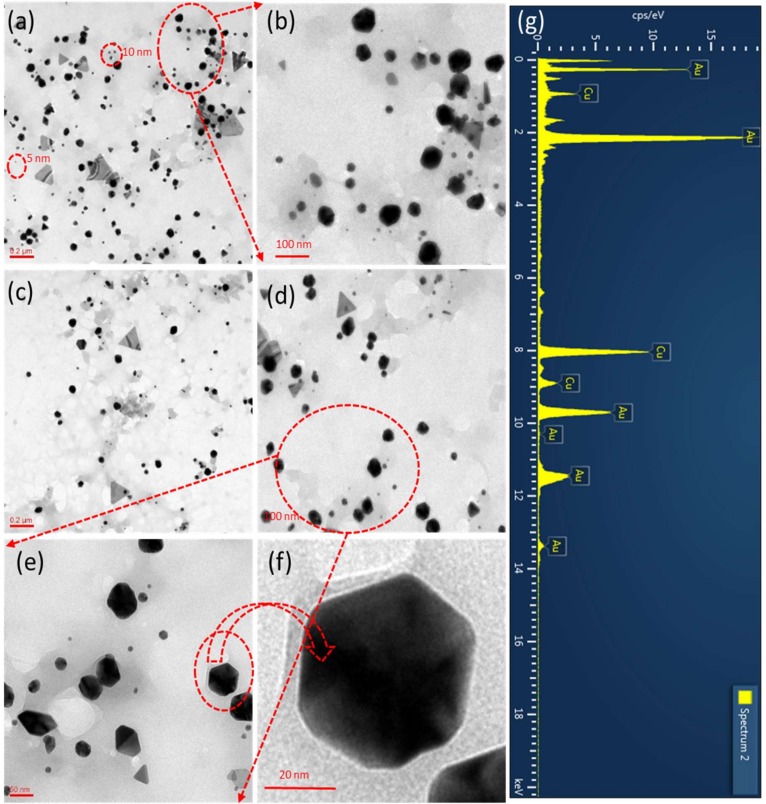
Transmission electron microscopy (TEM) images of green-synthesized hexagonal Au nanoparticles (AuNPs). (**a**) Overall view of sample. (**b**) Magnified image of area encircled in red in (**a**). (**c**) TEM image scanned at 200 nm. (**d**) Quasi-hexagonal AuNPs. (**e**) AuNPs scanned at 50 nm. (**f**) Magnified image of encircled AuNP in (**e**) that illustrates its hexagonal shape. (**g**) Energy-dispersive X-ray spectrum of hexagonal AuNPs.

The previous study revealed that the gold prisms (triangles) synthesized by lemongrass [31]. The reported gold prisms have around or more than 200 nm each side of prisms. Therefore, a method needs to develop to prepare small size. In our study, we have used *E. annuus* leaf extract for the synthesis of triangular and hexagonal AuNPs. The size and morphology of the as-synthesized AuNPs were examined using TEM. A carbon-coated Cu grid was used to prepare the samples for TEM analysis using the drop coating method. The TEM images in Figure 4a,c illustrate that the synthesized AuNPs presented triangular and hexagonal morphologies; the number of hexagonal AuNPs exceeded that of triangular ones. Using the overall TEM image of the sample (Figure 4a), which was scanned at 0.2 µm, we determined that the smallest AuNPs were approximately 5 nm in size (Figure 4a). Figure 4b (the magnified image of the area encircled in red in Figure 4a) illustrates the presence of quasi-hexagonal AuNPs in the sample, where the largest particles were 61 nm in size. Moreover, the TEM image in Figure 4a illustrates that most AuNPs were not spherical, except for a few very small ones.

**Figure 5:**
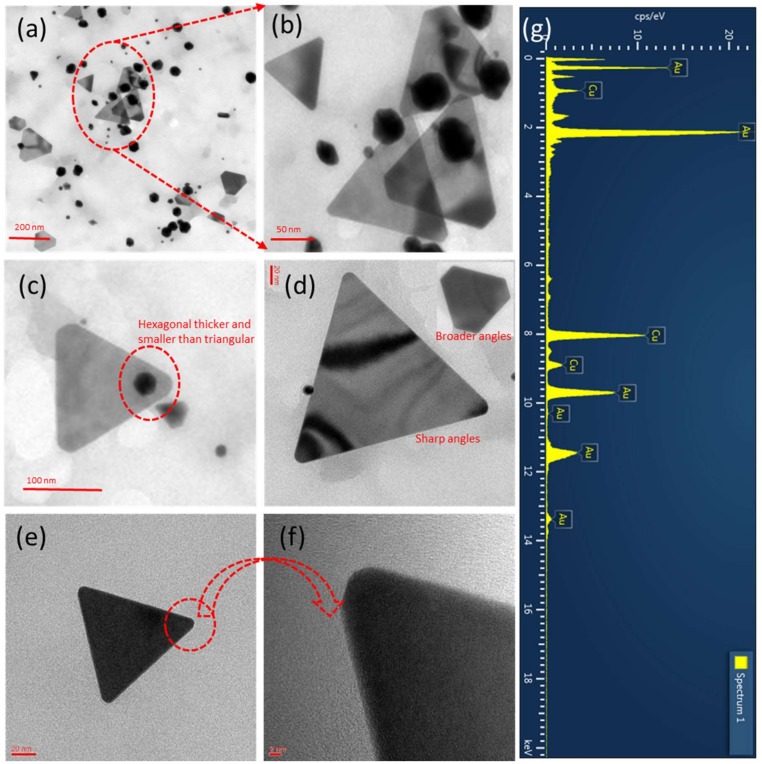
Transmission electron microscopy (TEM) images of green-synthesized triangular Au nanoparticles (AuNPs). (**a**) Overall view of sample. (**b**) Magnified image of area encircled in red in (**a**). (**c**) TEM image illustrating thickness difference between hexagonal and triangular AuNPs. (**d**) AuNPs featuring broad and sharp angles. (**e**) High-resolution transmission electron microscopy (HR-TEM) image of individual triangular AuNP. (**f**) HR-TEM image focused on angle feature of AuNP in (**e**). (**g**) Energy-dispersive X-ray spectrum of triangular AuNPs.

The TEM images in Figure 4c,d illustrate that the hexagonal green-synthesized AuNPs were larger than the spherical ones. When the particles were scanned at 50 nm (Figure 4e) it was determined that most particles were quasi-hexagonal in shape. Figure 4f (the magnified view of the area encircled in red in Figure 4e) illustrates the hexagonal morphology of the 47 nm AuNPs. According to the TEM images in Figure 5, the green-synthesized triangular AuNPs were equilateral in shape. The TEM image in Figure 5a illustrates two types of triangular AuNPs in the area encircled in red: sharp- and round-cornered ones. The sides of the smallest and largest triangular AuNPs were 23 and 178 nm, respectively. The darker the shade was of the triangular AuNPs, the thicker the sides were (Figure 5b). Figure 5b is the magnified TEM image of the area encircled in red in Figure 5a; this figure illustrates that the triangular AuNPs were very thin. Moreover, the triangular AuNPs and even stacks of two triangular AuNPs were thinner than the hexagonal AuNPs (Figure 5b,c). Furthermore, the TEM images in Figure 5d and its inset confirmed the presence of sharp- and round-cornered triangular AuNPs, respectively, in the sample. The TEM images in Figure 5e,f present a single triangular AuNP; its sides are 104 nm each and its corners are rounded. The TEM micrographs of green-synthesized triangular AuNPs were equilateral type.

### 3.5. EDX Analysis

The green-synthesized triangular and hexagonal AuNPs were characterized using EDX to determine their elemental composition. The EDX profiles were obtained during the TEM analysis of the AuNPs as the EDX device was an attachment of the TEM instrument. Figure 4g and Figure 5g depict the EDX profiles of hexagonal and triangular AuNPs, respectively, which include the strong signals of elemental Au. These signals revealed that the green-synthesized AuNPs consisted of elemental Au, as no other elemental signals were detected in the EDX profiles, except for Cu. The presence of the Cu signal was explained by the use of the Cu grid when preparing the samples. Hence, the synthesized triangular and hexagonal AuNPs consisted of pure elemental Au. Table 1 lists the elemental percentage, composition, series, and K-factor for the nanoparticles by the EDX.

### 3.6. Possible Green Synthesis Mechanism

The possible green synthesis mechanism of anionic AuNPs has been discussed to understand the formation. The polyphenolic compounds are present in the leaf extract which plays an important role in the synthesis of nanoparticles. *E. annuus* leaf extract is a prominent source of γ-pyranone derivatives, flavonoids, and phenolic acids [20], and these biomolecules show significant roles in the synthesis of NPs [5,21,22]. These polyphenolic compounds are abundantly existing in all parts of the plants which play a major role in counteracting the effect of reactive oxygen species (ROS) [5,22]. The flavonoids and tannins are abundantly found in aqueous extract of *E. annuus* leaf extract which shows high antioxidative activity. When leaf extract was added into gold(III) chloride trihydrate (HAuCl_4_·3H_2_O) solution, the reduction of Au^3+^ started into Au^0^. The neutralized Au0 undergoes physical phenomena nucleation to get its size and shape. FTIR analysis supported that the protein molecules stabilized the nanoparticles. An intense peak was witnessed at 1604.73 cm^−1^, which was attributed to the local environments of the COO^−^ groups in the protein molecule. The zeta potential of the synthesized nanoparticles was determined to be −26.7 mV, which indicated that the surface of the nanoparticles was negatively charged. The presence of COO^−^ groups (1604.73 cm^−1^) on the surface of nanoparticles is mainly responsible for the negative zeta potential [2]. The peak at 1070.46 cm^−1^, corresponded to the flavanones on the surface of the AuNPs. The FTIR analysis clearly revealed the participation of biological molecules for the synthesis of nanoparticles. However, further experimental analysis would be necessary to understand the detailed mechanism.

### 3.7. Effect of E. annuus Leaf Extract Concentration on Green Synthesis of AuNPs

**Figure 6:**
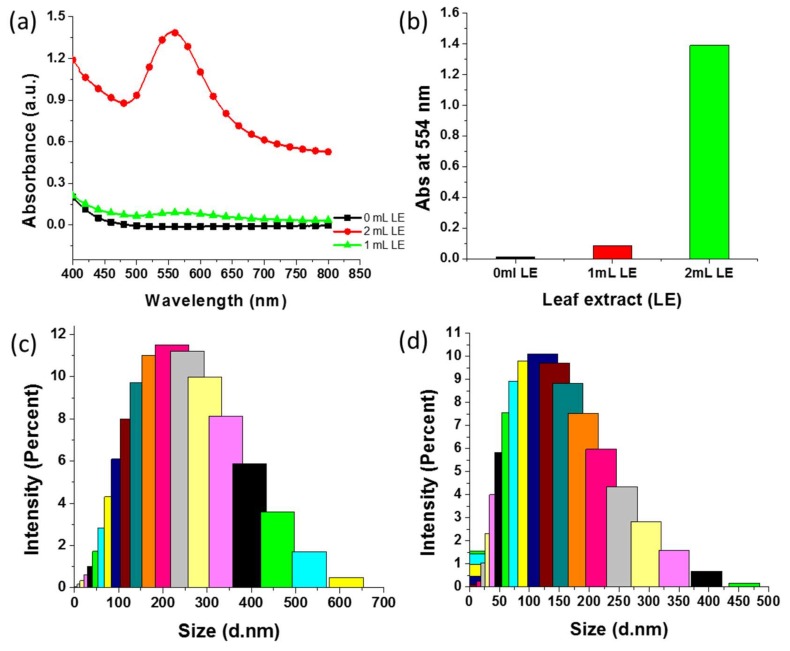
Effect of *Erigeron annuus* (*E. annuus*) leaf extract on synthesis of Au nanoparticles (AuNPs). (**a**) Ultraviolet–visible spectra of green-synthesized AuNPs using different leaf extract (LE) volumes. (**b**) Comparison of absorbance of reaction mixtures containing different volumes of *E. annuus* leaf extract at 554 nm. (**c**,**d**) Dynamic light scattering graphs of AuNPs synthesized using 1 and 2 mL *E. annuus* leaf extract, respectively.

The *E. annuus* leaf extract was used as both reducing and capping agents during the synthesis of the AuNPs [7]. Hence, the concentration of the leaf extract could play a significant role in the green synthesis process. The effect of the *E. annuus* leaf extract on the synthesis of AuNPs was analyzed using SPR as well as data on the size of the synthesized AuNPs. Figure 6a depicts the UV–Vis spectra of the AuNPs green-synthesized using different *E. annuus* leaf extract concentrations. No reaction occurred in the absence of the leaf extract, as the color of the reactant mixture remained unchanged as time went by and no peaks were observed in the UV–Vis spectrum of the mixture. When the HAuCl_4_·3H_2_O solution was treated with 1 mL *E. annuus* leaf extract, the color of the solution changed from light yellow to light red and a very weak peak was observed in the UV–Vis spectrum. Whereas, when 2 mL *E. annuus* leaf extract was added to the HAuCl_4_·3H_2_O solution, the color of the final mixture was bright red. The SPR of the AuNPs synthesized using 2 mL *E. annuus* leaf extract was stronger than that of the AuNPs synthesized using 1 mL leaf extract (Figure 6a). Moreover, the absorbance of the reaction mixture that contained 2 mL *E. annuus* leaf extract was higher than that of the mixture that contained 1 mL leaf extract at 554 nm (Figure 6b). The sizes of the AuNPs synthesized using 1 and 2 mL *E. annuus* leaf extract were analyzed using DLS (Figure 6c,d, respectively). Using these size distribution profiles, the Z-average sizes of the AuNPs synthesized using 1 and 2 mL *E. annuus* leaf extract were determined to be 200 and 100 nm, respectively. Therefore, 2 mL was determined to be the optimum *E. annuus* leaf extract volume for the synthesis of AuNPs; the AuNPs obtained using 2 mL leaf extract presented the smallest size and highest absorbance (highest AuNP yield) of all synthesized AuNPs in this study. 

### 3.8. Effect of Reaction Temperature on Green Synthesis of AuNPs

**Figure 7:**
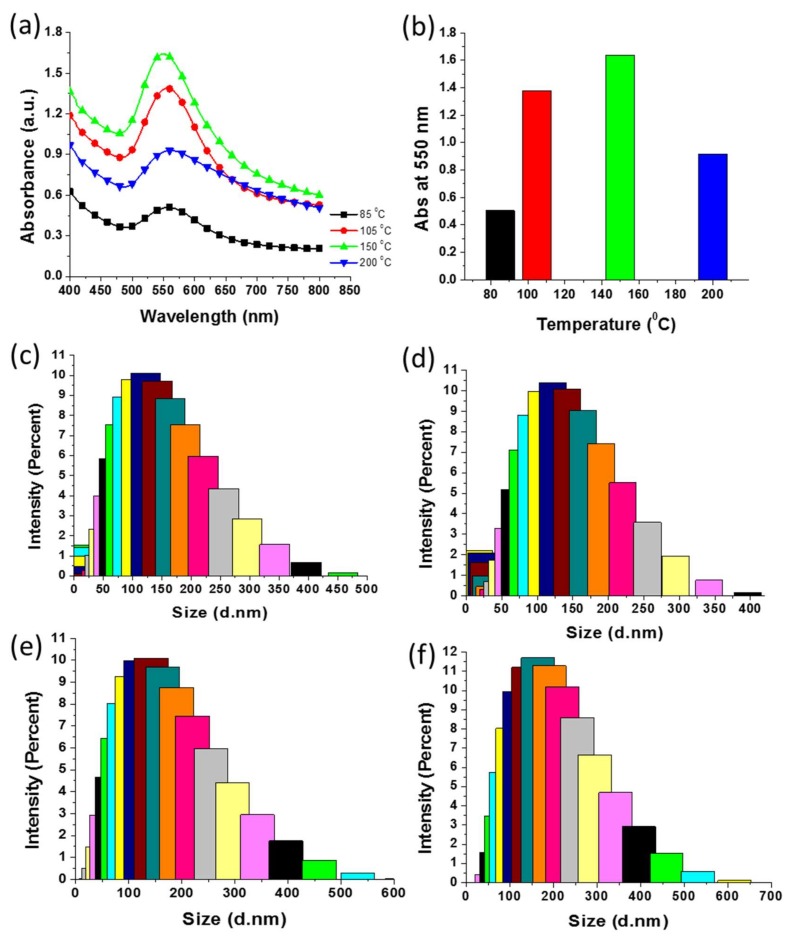
Effect of temperature on green synthesis of Au nanoparticles (AuNPs). (**a**) Ultraviolet–visible spectra of AuNPs green-synthesized at different temperatures. (**b**) Comparison of absorbance of AuNPs synthesized at different temperatures at 550 nm. (**c**–**f**) Dynamic light scattering graphs of AuNPs synthesized at 85, 105, 150, and 200 °C, respectively.

The effects of the temperature on the SPR and size of AuNPs were evaluated using UV–Vis spectroscopy and DLS, respectively. Agarwal et al. indicated that temperature is an important parameter for nanoparticle synthesis [11]. We synthesized AuNPs at various temperatures in the range 85–200 °C using 2 mL *E. annuus* leaf extract. The UV–Vis spectra of the reaction mixtures revealed that all temperatures supported the green synthesis of AuNPs as SPR was observed at approximately 550 nm regardless of the temperature (Figure 7a). The absorbance increased as the synthesis temperature increased from 85 to 105 and 150 °C, but decreased as the temperature was further increased to 200 °C (Figure 7b). The highest absorbance was observed when the reaction was performed at 150 °C. The effect of the temperature on the size of the AuNPs was also determined using DLS. Figure 7c,d illustrates that the Z-average values of the AuNPs synthesized at 85, 105, 150, and 200 °C were approximately 100, 120, 110, and 150 nm, respectively. Therefore, the results supported the hypothesis that increasing the synthesis temperature increased the Z-average size of the AuNPs. 

### 3.9. Cytotoxicity Assays

**Figure 8:**
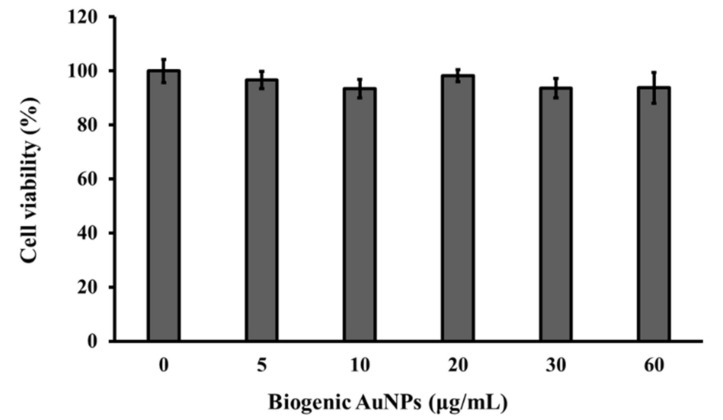
Cell viability assay in presence of different concentrations of biogenic Au nanoparticles (AuNPs) against 3T3-L1 adipocytes.

The cytotoxicity of the green-synthesized AuNPs was examined against 3T3-L1 adipocytes. Various concentrations of AuNPs in the 5–60 µg/mL range were supplemented with DMEM that contained 10% FBS and antibiotic-antimycotic solution. The cell viability, expressed as a percentage of untreated control adipocytes (100% cell viability) was examined using EZ-Cytox assay kits. After analyzing the assays for 48 h, it was concluded that the green-synthesized AuNPs did not affect the viability of the 3T3-L1 adipocytes, even at the high concentration of 60 µg/mL (Figure 8). Vijayakumar and Ganesan indicated that citrate-stabilized AuNPs (chemically synthesized) considerably affected cell viability compared with biologically stable AuNPs [32]. Using cell viability data, we concluded that the AuNPs synthesized using *E. annuus* leaf extract did not exhibit any adverse effects on 3T3-L1 adipocytes. However, a very slightly negative slope was found which represents very less cytotoxic at high concentration of nanoparticles. The previous study has used very few concentration gold nanoparticles for cytotoxicity analysis [33,34]. We have found that cytotoxicity is very less with a high concentration of nanoparticles. It was observed that the 93.83% cell viability was found after 48 h incubation. Figure 8 does not show a sharp decrease in the cell viability with increasing concentration of nanoparticles. Therefore, our present study showed that the synthesized nanoparticles were no-toxic or very less toxic to 3T3-L1 adipocytes.

## 4. Conclusions

In this study, we have developed an environmentally friendly method for the synthesis of triangular and hexagonal AuNPs. The method did not require the use of hazardous chemicals or any chemicals except for Au salt. *E. annuus* leaf extract, which acted as a reducing as well as a capping agent. Further, this extract was used for the synthesis of triangular and hexagonal AuNPs. The surface potential of the green-synthesized AuNPs was negative and could be easily modified. The TEM images of the AuNPs illustrated that a fraction of the synthesized AuNPs was quasi-hexagonal, and the largest quasi-hexagonal AuNPs were 61 nm in size. In addition, two types of equilateral triangular AuNPs were synthesized—sharp- and round-cornered—as observed in the TEM images that are included in the manuscript. The strong signals of Au in the EDX profiles of the triangular and hexagonal AuNPs revealed that elemental Au was the only element present in the synthesized AuNPs. Furthermore, the green-synthesized AuNPs did not present cytotoxic effects on 3T3-L1 adipocytes; this was evidenced using cell viability analysis.

## Figures and Tables

**Table 1:** Elemental composition of Au nanoparticles acquired using energy-dispersive X-ray spectroscopy.

Element	Line Type	K Factor	Absorption Correction	Wt.%	Wt.% Sigma	At.%
Cu	K series	1.233	0.52	28.31	0.30	55.03
Au	L series	2.265	0.52	71.69	0.30	44.97
Total				100.00		100.00
